# Supplementary material for: Determination of Whether Apex or Non-Apex Prostate Cancer Is the Best Candidate for the Use of Prostate-Specific Antigen Density to Predict Pathological Grade Group Upgrading and Upstaging after Radical Prostatectomy
Source: J Clin Med. 2023 Feb 19;12(4):1659. doi: 10.3390/jcm12041659 (PMC9967179; doi:10.3390/jcm12041659)
Supplement: Supplementary file 1 [file jcm-12-01659-s001.zip › jcm-2171713-supplementary.pdf]

**Table S1.** Comparison of upgrading and upstaging in all of patients (n = 535).

| Variable | No-upgrading (n=290, 54.2%) | upgrading (n=245, 45.8%) | *p* value | No-upstaging (n=273, 51.0%) | Upstaging (n=262, 49.0%) | *p* value |
|---|---|---|---|---|---|---|
| **Age, years** | | | 0.205 | | | 0.359 |
| Median (IQR) | 67 (61-71) | 67 (62-71) | | 67 (62-71) | 67 (61.75-72) | |
| Mean ± SD | 65.76 ± 6.77 | 66.56 ± 6.45 | | 65.85 ± 6.65 | 66.41 ± 6.63 | |
| **BMI, kg/m$^2$** | | | 0.418 | | | 0.110 |
| Median (IQR) | 24.34 (22.70-26.30) | 24.22 (22.60-26.12) | | 24.22 (22.39-26.04) | 24.49 (22.87-26.56) | |
| Mean ± SD | 24.61 ± 2.88 | 24.37 ± 2.86 | | 24.31 ± 2.86 | 24.70 ± 2.87 | |
| **Serum PSA, ng/ml** | | | **< 0.001*** | | | **< 0.001*** |
| Median (IQR) | 9.73 (6.95-15.42) | 12.33 (8.47-19.42) | | 9.16 (6.73-13.60) | 12.75 (8.66-20.17) | |
| Mean ± SD | 13.31 ± 12.43 | 15.86 ± 11.68 | | 12.33 ± 11.14 | 16.71 ± 12.76 | |
| **Prostate volume, ml** | | | 0.119 | | | **< 0.001*** |
| Median (IQR) | 40.80 (30.00-59.63) | 38.80 (29.41-53.00) | | 44.10 (32.20-65.00) | 36.00 (27.50-48.74) | |
| Mean ± SD | 48.64 ± 27.09 | 44.58 ± 22.92 | | 52.23 ± 28.51 | 41.11 ± 20.04 | |
| **PSAD, ng/ml$^2$** | | | **< 0.001*** | | | **< 0.001*** |
| Median (IQR) | 0.22 (0.14-0.36) | 0.32 (0.20-0.52) | | 0.20 (0.13-0.32) | 0.34 (0.22-0.54) | |
| Mean ± SD | 0.31 ± 0.30 | 0.42 ± 0.35 | | 0.28 ± 0.27 | 0.45 ± 0.35 | |
| **Number of biopsy cores** | | | 0.430 | | | 0.809 |
| Median (IQR) | 13 (12-14) | 13 (12-14) | | 13 (12-14) | 13 (12-14) | |
| Mean ± SD | 13.32 ± 2.37 | 13.16 ± 2.44 | | 13.33 ± 2.62 | 13.16 ± 2.17 | |
| **Number of positive cores** | | | 0.376 | | | **< 0.001*** |
| Median (IQR) | 4 (2-7) | 4 (2-6) | | 3 (1-5) | 5 (3-8) | |
| Mean ± SD | 4.55 ± 3.05 | 4.34 ± 3.09 | | 3.57 ± 2.58 | 5.38 ± 3.26 | |
| **Percent positive biopsy cores, %** | | | 0.599 | | | **< 0.001*** |

| | | | | | | |
|---|---|---|---|---|---|---|
| Median (IQR) | 31.41 (14.29-50.00) | 28.57 (15.38-46.67) | | 23.08 (8.33-39.44) | 38.46 (20.00-56.77) | |
| Mean ± SD | 34.92 ± 24.11 | 33.20 ± 23.11 | | 27.18 ± 19.61 | 41.36 ± 25.31 | |
| **Max core involvement, %** | | | 0.393 | | | **< 0.001*** |
| Median (IQR) | 70.0 (33.0-85.0) | 60.0 (30.0-85.0) | | 50.0 (20.0-85.0) | 80.0 (50.0-85.0) | |
| Mean ± SD | 59.91 ± 29.76 | 57.23 ± 30.69 | | 51.19 ± 30.38 | 66.49 ± 27.98 | |

IQR, interquartile range; SD, standard deviation; BMI, body mass index; PSA, prostate-specific antigen; DRE, digital rectal examination; PSAD; prostate-specific antigen density. *statistically significant.

**Table S2.** Comparison of upgrading and upstaging in patients with NAPCa (n = 374).

| Variable | No-upgrading (n=206, 55.1%) | upgrading (n=168, 44.9%) | *p* value | No-upstaging (n=215, 57.5%) | Upstaging (n=159, 42.5%) | *p* value |
|---|---|---|---|---|---|---|
| **Age, years** | | | 0.506 | | | 0.694 |
| Median (IQR) | 66 (61.75-71) | 67 (62-71) | | 67 (62-71) | 66 (62-71) | |
| Mean ± SD | 65.89 ± 6.60 | 66.46 ± 6.03 | | 65.99 ± 6.36 | 66.36 ± 6.34 | |
| **BMI, kg/m²** | | | 0.423 | | | 0.293 |
| Median (IQR) | 24.22 (22.27-26.21) | 24.22 (22.32-25.61) | | 24.22 (22.05-25.95) | 24.22 (22.86-26.31) | |
| Mean ± SD | 24.39 ± 2.87 | 24.13 ± 2.89 | | 24.13 ± 2.92 | 24.46 ± 2.83 | |
| **Serum PSA, ng/ml** | | | **0.001*** | | | **< 0.001*** |
| Median (IQR) | 9.20 (6.67-14.97) | 11.83 (7.94-17.83) | | 8.98 (6.36-14.79) | 11.85 (8.12-18.17) | |
| Mean ± SD | 12.16 ± 10.92 | 14.46 ± 10.30 | | 12.16 ± 11.30 | 14.59 ± 9.66 | |
| **Prostate volume, ml** | | | 0.068 | | | **< 0.001*** |
| Median (IQR) | 40.00 (29.93-60.45) | 37.70 (28.00-51.70) | | 43.95 (31.40-60.15) | 35.30 (25.00-44.15) | |
| Mean ± SD | 49.18 ± 29.18 | 43.25 ± 22.76 | | 50.91 ± 29.84 | 37.61 ± 18.75 | |
| **PSAD, ng/ml²** | | | **< 0.001*** | | | **< 0.001*** |
| Median (IQR) | 0.21 (0.12-0.34) | 0.31 (0.21-0.50) | | 0.18 (0.11-0.30) | 0.35 (0.24-0.59) | |
| Mean ± SD | 0.28 ± 0.27 | 0.43 ± 0.36 | | 0.24 ± 0.24 | 0.48 ± 0.38 | |
| **Number of biopsy cores** | | | 0.416 | | | 0.574 |
| Median (IQR) | 13 (12-14) | 13 (12-15) | | 13 (12-15) | 13 (12-14.75) | |
| Mean ± SD | 13.77 ± 2.80 | 13.86 ± 3.06 | | 13.95 ± 2.28 | 13.63 ± 2.39 | |
| **Number of positive cores** | | | 0.414 | | | **< 0.001*** |
| Median (IQR) | 5 (2-7) | 4 (2-6) | | 3 (1-6) | 5.5 (3-8) | |
| Mean ± SD | 4.81 ± 2.97 | 4.20 ± 2.73 | | 3.76 ± 2.63 | 5.52 ± 2.88 | |
| **Percent positive biopsy cores, %** | | | 0.610 | | | **< 0.001*** |
| Median (IQR) | 33.33 (14.29-50.00) | 27.62 (14.56-43.53) | | 23.08 (8.52-40.00) | 39.69 (25.00-53.85) | |

| | | | | | | |
|---|---|---|---|---|---|---|
| Mean ± SD | 36.00 ± 23.41 | 30.83 ± 20.14 | | 27.81 ± 20.20 | 41.16 ± 22.29 | |
| **Max core involvement, %** | | | 0.324 | | | **0.007*** |
| Median (IQR) | 70.0 (35.0-85.0) | 50.0 (20.0-70.0) | | 50.0 (20.0-75.25) | 70.0 (40.0-85.0) | |
| Mean ± SD | 59.34 ± 29.27 | 50.20 ± 30.22 | | 50.27 ± 30.46 | 61.63 ± 28.25 | |

NAPCa, non-apex prostate cancer; IQR, interquartile range; SD, standard deviation; BMI, body mass index; PSA, prostate-specific antigen; DRE, digital rectal examination; PSAD; prostate-specific antigen density. *statistically significant.

**Table S3.** Comparison of upgrading and upstaging in patients with APCa (n = 161).

| Variable | No-upgrading (n=84, 52.2%) | upgrading (n=77, 47.8%) | *p* value | No-upstaging (n=58, 36.0%) | Upstaging (n=103, 64.0%) | *p* value |
|---|---|---|---|---|---|---|
| **Age, years** | | | 0.215 | | | 0.384 |
| Median (IQR) | 67 (60.25-71) | 68 (62-72) | | 67 (62-71) | 67 (62-71) | |
| Mean ± SD | 65.44 ± 7.22 | 66.77 ± 7.36 | | 65.36 ± 7.65 | 66.48 ± 7.09 | |
| **BMI, kg/m$^2$** | | | 0.647 | | | 0.665 |
| Median (IQR) | 24.61 (23.38-26.79) | 24.86 (22.90-26.66) | | 24.47 (23.38-26.47) | 24.91 (23.03-27.06) | |
| Mean ± SD | 24.61 ± 2.88 | 24.37 ± 2.86 | | 24.98 ± 2.57 | 25.08 ± 2.90 | |
| **Serum PSA, ng/ml** | | | **0.024*** | | | **< 0.001*** |
| Median (IQR) | 10.86 (7.74-16.83) | 13.30 (9.44-25.09) | | 9.38 (7.55-13.02) | 13.89 (9.51-24.86) | |
| Mean ± SD | 16.13 ± 15.28 | 18.95 ± 13.86 | | 12.97 ± 10.59 | 20.00 ± 15.97 | |
| **Prostate volume, ml** | | | 0.929 | | | 0.146 |
| Median (IQR) | 42.28 (30.78-56.75) | 41.40 (31.05-60.88) | | 45.90 (33.60-67.30) | 41.30 (30.00-57.00) | |
| Mean ± SD | 47.34 ± 21.22 | 47.47 ± 23.17 | | 50.83 ± 23.76 | 45.47 ± 20.99 | |
| **PSAD, ng/ml$^2$** | | | 0.182 | | | **0.002*** |
| Median (IQR) | 0.25 (0.18-0.44) | 0.35 (0.18-0.55) | | 0.23 (0.16-0.30) | 0.35 (0.22-0.54) | |
| Mean ± SD | 0.39 ± 0.35 | 0.42 ± 0.31 | | 0.31 ± 0.29 | 0.43 ± 0.33 | |
| **Number of biopsy cores** | | | 0.900 | | | 0.237 |
| Median (IQR) | 13 (12-13) | 13 (12-14) | | 12 (12-13) | 13 (12-14) | |
| Mean ± SD | 13.21 ± 2.37 | 12.86 ± 1.77 | | 13.03 ± 2.01 | 13.05 ± 2.17 | |
| **Number of positive cores** | | | 0.633 | | | **0.007*** |
| Median (IQR) | 5 (3-7.75) | 5 (2-7) | | 4 (2-6) | 6 (3-8) | |
| Mean ± SD | 5.37 ± 3.23 | 5.17 ± 3.32 | | 4.29 ± 2.73 | 5.83 ± 3.42 | |
| **Percent positive biopsy cores, %** | | | 0.764 | | | **0.005*** |
| Median (IQR) | 38.46 (20.56-53.85) | 37.50 (16.67-53.85) | | 30.77 (16.67-42.79) | 42.86 (22.22-69.23) | |

| | | | | | | |
|---|---|---|---|---|---|---|
| Mean ± SD | 41.44 ± 25.20 | 40.13 ± 37.50 | | 32.82 ± 20.01 | 45.32 ± 26.54 | |
| **Max core involvement, %** | | | 0.642 | | | **0.005*** |
| Median (IQR) | 85.0 (42.5-85.0) | 85.0 (50.0-85.0) | | 65.0 (30.0-85.0) | 85.0 (50.0-85.0) | |
| Mean ± SD | 66.13 ± 28.23 | 65.25 ± 28.70 | | 57.59 ± 30.09 | 70.28 ± 26.42 | |

APCa, apex prostate cancer; IQR, interquartile range; SD, standard deviation; BMI, body mass index; PSA, prostate-specific antigen; DRE, digital rectal examination; PSAD; prostate-specific antigen density. *statistically significant.
